# Effect of temperature up-shift on fermentation and metabolic characteristics in view of gene expressions in *Escherichia coli*

**DOI:** 10.1186/1475-2859-7-35

**Published:** 2008-12-02

**Authors:** Chowdhurry Mohammad Monirul Hasan, Kazuyuki Shimizu

**Affiliations:** 1 Department of Bioscience & Bioinformatics, Kyushu Institute of Technology, Iizuka, Fakuoka 820-8502, Japan; 2 Institute of Advanced Bioscience, Keio University, Tsuruoka, Yamagata 997-0017, Japan

## Abstract

**Background:**

*Escherichia coli *induces heat shock genes to the temperature up-shift, and changes the metabolism by complicated mechanism. The heat shock response is of practical importance for the variety of applications such as temperature-induced heterologous protein production, simultaneous saccharification and fermentation (SSF) etc. However, the effect of heat shock on the metabolic regulation is not well investigated. It is strongly desired to understand the metabolic changes and its mechanism upon heat shock in practice for the efficient metabolite production by temperature up-shift. In the present research, therefore, we investigated the effect of temperature up-shift from 37°C to 42°C on the metabolism in view of gene expressions.

**Results:**

The results of aerobic batch and continuous cultivations of *E. coli *BW25113 indicate that more acetate was accumulated with lower biomass yield and less glucose consumption rate at 42°C as compared to the case at 37°C. The down- regulation of the glucose uptake rate corresponds to the down-regulation of *ptsG *gene expression caused by the up-regulation of *mlc *gene expression. In accordance with up-regulation of *arcA*, which may be caused by the lower oxygen solubility at 42°C, the expressions of the TCA cycle-related genes and the respiratory chain gene *cyoA *were down-regulated. The decreased activity of TCA cycle caused more acetate formation at higher temperature, which is not preferred in heterologous protein production etc. This can be overcome by the *arcA *gene knockout to some extent. The time courses of gene expressions revealed that the heat shock genes such as *groEL, dnaK, htpG *and *ibpB *as well as *mlc *were expressed in much the same way as that of *rpoH *during the first 10–20 minutes after temperature up-shift. Under microaerobic condition, the fermentation changed in such a way that formate and lactate were more produced due to up-regulation of *pflA *and *ldhA *genes while ethanol was less produced due to down-regulation of *adhE *gene at higher temperature as compared to the case at 37°C.

**Conclusion:**

The present result clarified the mechanism of metabolic changes upon heat shock from 37°C to 42°C based on gene expressions of heat shock genes, global regulators, and the metabolic pathway genes. It is recommended to use *arcA *gene knockout mutant to prevent higher acetate production upon heat shock, where it must be noted that the cell yield may be decreased due to TCA cycle activation by *arcA *gene knockout.

## Background

Biological systems are known to be robust and adaptable to the culture environment. It became apparent that such robustness is inherent in the biochemical and genetic networks. Several genes that are necessary to respond to various environmental or nutritional changes require specific recognition by RNA polymerase associated with the alternative sigma factors such as σ^32 ^[1], σ^E ^[2], σ^54 ^[3], and σ^S ^[4]. In particular, the organisms respond to a sudden temperature up-shift by increasing the synthesis of a set of proteins. This phenomenon is called the heat shock response. The research on heat shock response of a microorganism contributes to the variety of practical applications such as temperature-induced heterologous protein production [5,6], simultaneous saccharification and fermentation (SSF) [7] etc.

The heat shock proteins play roles in the assembly and disassembly of macromolecular complex such as GroE [8], the intracellular transport such as Hsp70 [9], transcription such as σ^70 ^[10], proteolysis such as Lon [11], and translation such as lysil tRNA synthetase [12]. The heat shock response in *E. coli *is mediated by Eσ^32 ^[13], and it has been known that at least 26 genes are induced by heat shock [14], where E denotes the RNA polymerase holoenzyme. Among them, *groEL, dnaK*, and *htpG *are the genes which code for the major chaperones such as Hsp 60, Hsp 70, and Hsp 90. The ClpP, Lon and HtrC are involved in proteolysis. *DnaK, DnaJ*, and *GrpE *and *RpoH *are involved in the autoregulation of heat shock response [15-18]. It has been known that DnaK prevents the formation of inclusion bodies by reducing aggregation and promotion of proteolysis of misfolded proteins [19]. A bi-chaperone system involving DnaK and ClpB mediates the solubilization or disaggregation of proteins [20]. GroEL operates protein transit between soluble and unsoluble protein fractions and participates positively in disaggregation and inclusion body formation. Small heat shock proteins such as IbpA and IbpB protect heat- denatured proteins from irreversible aggregation and have been found to be associated with inclusion bodies [21,22].

Although many papers have been reported on the molecular mechanisms of heat shock proteins [23,24], very few researches have been reported on the effect of heat shock on the metabolism. Hoffmann et al. [6] investigated the metabolic adaptation of *E. coli *during temperature-induced recombinant protein production, and showed that cAMP/Crp-controlled LpdA of the pyruvate dehydrogenase complex(PDHc) and SdhA in the TCA cycle were induced four times, reaching a maximum at 1 h after temperature up-shift. It was also shown that the TCA cycle enzymes such as IcdA and Mdh were initially less produced but regained to their respective preshift values about 30 min after the temperature up-shift. More recently, Gadgil et al. [25] investigated the effect of temperature down-shift from 37°C to 33 and 28°C on gene expressions in *E. coli*. This kind of investigation is useful in analyzing the metabolic changes and investigating the effects of gene modification for strain improvement [26].

In the present study, we investigated how gene expression pattern changes in *E. coli *for the temperature up-shift from 37 to 42°C in relation to fermentation characteristics. The genes considered in the present study are listed in Additional file 1. The global regulators and their regulated genes are summarized in Additional file 2. Moreover, we investigated the dynamics of the expressions of such genes as heat shock genes, global regulators, and metabolic pathway genes to clarify the metabolic regulation upon heat shock.

## Results

The batch cultivation results at two different temperatures of 37°C and 42°C indicate that the specific glucose consumption rate was lower at 42°C than at 37°C (data not shown). The maximum acetate concentration was 2.91 g/l at 42°C. Acetate was little consumed even after the glucose was depleted, whereas maximum acetate concentration was 2.69 g/l at 37°C. Acetate was consumed after the glucose was depleted at 37°C. The final cell concentration was lower at 42°C as compared to that at 37°C (3.32 and 3.92 g/l, respectively).

In order to make clear this phenomenon, aerobic continuous cultivation was conducted at the dilution rate of 0.2 h^-1^, where Table 1 shows the effect of culture temperature on the fermentation parameters. Table 1 indicates that acetate was more accumulated (p < 0.05), the cell yield was lower (p < 0.05), and the specific glucose consumption rate was lower (p < 0.1) at 42°C as compared to the case at 37°C, which is consistent with the above mentioned batch data.

**Table 1:** Fermentation parameters for the aerobic chemostat culture of the wild type *E. coli *BW25113 at the dilution rate of 0.2 h^-1^

Fermentation parameters	Culture temperature		% changes
		
	**37°C**	**42°C**	
Specific glucose uptake rate (mmol/gDCW/h)	2.46 ± 0.05	2.28 ± 0.04	- 7.31
Specific acetate production rate (mmol/gDCW/h)	0.18 ± 0.02	0.32 ± 0.04	+77.8
Specific CER (mmol/gDCW/h)	5.83 ± 0.05	5.87 ± 0.04	+ 0.69
Specific OUR (mmol/gDCW/h)	5.74 ± 0.06	5.76 ± .04	+ 0.35
Biomass yield (gDCW/g substrate	0.42 ± 0.01	0.34 ± 0.02	- 19.0

± Standard deviation of three independent experiments.

**Figure 1:**
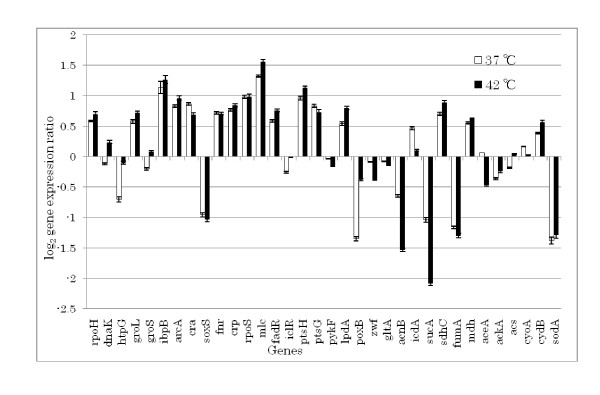
Effect of temperature up-shift on gene expressions in *E. coli *BW25113 under aerobic continuous culture at the dilution rate of 0.2 h^-1 ^(a) comparison of gene expressions (The gene expressions are given as relative values to that of *dnaA*) (b) change in gene expressions on the metabolic pathways (red represents up-regulation, blue represents down-regulation, and black represents no change).

Figure 1a compares the gene expressions for the two different culture temperatures in the continuous culture (Table 1), where Fig. 1b depicts the changes in gene expressions on the metabolic pathways. Note that the samples were taken after the steady state was confirmed after the temperature was raised to 42°C. Figure 1 a indicates that the expression of *rpoH *was up-regulated (p < 0.10), and the expressions of *dnaK, groL, groS, htpG*, and *ibpB *were up-regulated (p < 0.1, p < 0.1, p < 0.05, p < 0.05 and p < 0.05, respectively), which are known to be under control of sigma factor (σ^32^). Figure 1 also shows the up-regulation of *arcA *gene expression (p < 0.1), where *arcA *gene product functions as a repressor of such genes as involved in the TCA cycle under microaerobic condition (Additional file 2) [27]. Figure 1 indicates that some of the TCA cycle and glyoxylate pathway genes such as *icdA*, *sucA *and *aceA *were down-regulated (p < 0.1, p < 0.1 and p < 0.05, respectively). Those genes are known to be under control of ArcA (*arcA *gene product) (Additional file 2). Figure 1 also indicates that the expressions of the respiratory chain genes such as *cydB *was up-regulated (p < 0.1), whereas *cyoA *was down regulated (p < 0.1). This may be due to the up-regulation of *arcA *and its gene product ArcA, since *cydB *operon is under the positive control, while *cyoA *is under negative control of ArcA (Additional file 2). Figure 1 also indicates that the expression of *crp *gene, which codes for cAMP receptor protein Crp, was up-regulated (p < 0.1), and the expression of *lpdA*, which is known to be under control of Crp (Additional file 2), was also up-regulated (p < 0.1) [6]. Moreover, *mlc *(making larger colonies) gene expression was higher, and the *ptsG *gene expression was lower (p < 0.05). Figure 1 also shows that *cra *(catabolite repressor activator) gene expression was down-regulated (p < 0.1), where *cra *gene product regulates the carbon flow in such a way that the gluconeogenesis was activated, whereas the glycolysis was repressed (Additional file 2). The gene expressions of *fadR *and *iclR *were also higher, where FadR activates *iclR*, and IclR is known to repress *aceBAK*.

**Figure 2:**
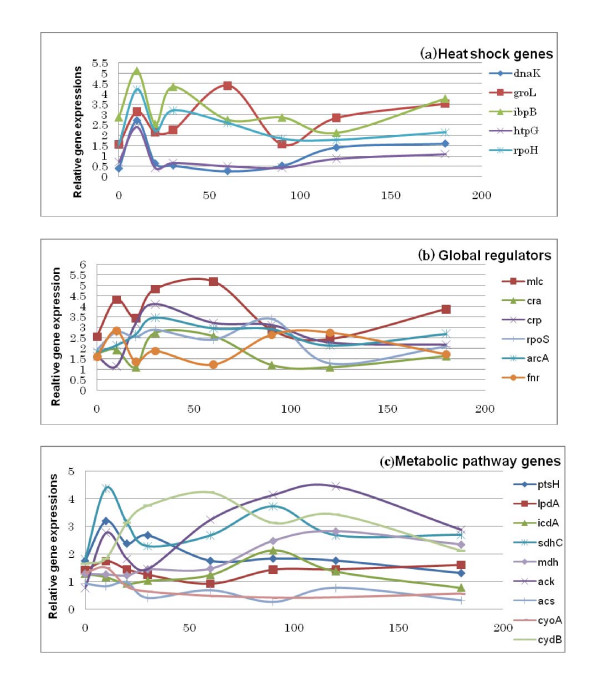
Responses of gene expressions of *E. coli *BW25113 for the temperature up-shift (37°C to 42°C) under aerobic continuous culture at the dilution rate of 0.2 h^-1^. heat shock genes (b) global regulators (c) metabolic pathway genes.

It has been known that the gene network forms a circuit so that the metabolic system is robust against external stimuli such as heat shock. Figure 2a shows how *rpoH *and the heat shock protein genes responded to the temperature up-shift with respect to time. The expression of *rpoH *gene shows an overshoot phenomenon during the initial 10–20 min, where this may be due to the feedback in the gene network. Moreover, the expressions of *dnaK, groL, ibpB, htpG *followed the same pattern as that of *rpoH*, which implies that those genes are under control of *rpoH *or σ^32^.

Figure 2b shows the dynamic behavior of the global regulators, where it indicates that the expressions of *mlc, crp, arcA *tended to increase after the temperature up-shift, where it has been reported that the promoter regions of *mlc *and *crp *genes have the binding site for *rpoH *[28,29], whereas there is no such report on *arcA*.

Figure 2c shows the dynamic behavior of the metabolic pathway gene expressions, where *ptsH *and *sdhC *expressions became high at 10 min after the temperature up-shift but eventually settled down to lower values. Although *sdhC *gene expression shows little change in Fig. 1, Fig. 2c shows an increase in its expression level during the first 10 minutes after the temperature up-shift [6]. On the other hand, other TCA cycle genes such as *mdh *and *icdA *showed decrease expression during the first 30 mins [6]. The *ackA *gene expression tended to increase while *acs *expression tended to decrease, which may have partly caused the acetate accumulation. The expression of the respiratory chain gene such as *cydB *increased, while *cyoA *gene expression decreased.

Figures 1 and 2 indicate that *arcA *gene expression increased (p < 0.1), and the expressions of such genes as those under control of ArcA (Additional file 2) changed accordingly. Since *arcA *is not known to be under control of *rpoH*, the change in *arcA *gene expression may be indirect due to the lower dissolved oxygen concentration caused by the temperature up-shift. In order to see this in more detail, we cultivated *arcA *gene knockout mutant. Table 2 shows the fermentation parameters for the continuous cultivations of *arcA *mutant at two different temperatures. If we compare the results of Tables 1 and 2, the specific glucose consumption rate was higher, the specific acetate production rate was lower, and the cell yield was lower for the case of *arcA *mutant as compared to each case of 37°C and 42°C of the wild type strain. Table 2 also indicates that the acetate production rate was higher at 42°C as compared to the case at 37°C, where similar trend can be seen in Table 1.

**Table 2:** Fermentation parameters for the aerobic chemostat culture of *arcA *mutant at the dilution rate of 0.2 h^-1^

Fermentation parameters	Culture temperature		% changes
		
	**37°C**	**42°C**	
Specific glucose uptake rate (mmol/gDCW/h)	2.62 ± 0.06	2.48 ± 0.04	- 5.34
Specific acetate production rate (mmol/gDCW/h)	0.11 ± 0.05	0.16 ± 0.07	+ 45.5
Specific CER (mmol/gDCW/h)	5.98 ± 0.05	6.22 ± 0.04	+ 4.01
Specific OUR (mmol/gDCW/h)	5.87 ± 0.06	6.11 ± 0.05	+ 4.08
Biomass yield (gDCW/g substrate	0.40 ± 0.01	0.35 ± 0.02	- 12.5

± Standard deviation of three independent experiments.

**Figure 3:**
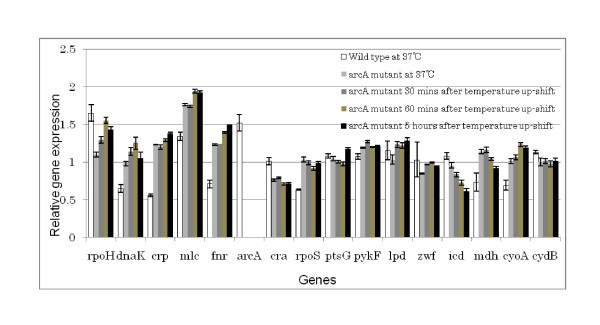
Effect of temperature on gene expressions of *arcA *gene knockout mutant under aerobic continuous culture at the dilution rate of 0.2 h^-1^.

Figure 3 shows how gene expressions of *arcA *mutant changed at 30 min, 60 min, and 5 hours after temperature up-shift as compared to the case at 37°C as well as those of the wild type strain (white bar). By comparing the gene expressions of the wild type and *arcA *mutant at 37°C, it can be seen that *mdh *and *cyoA *genes were up-regulated, while *cydB *gene was down-regulated for *arcA *mutant. Figure 3 also indicates that *dnaK *gene expression followed the same time profile as that of *rpoH*. The *crp *and *mlc *gene expressions became higher as time proceeded. Figure 3 also indicates that *fnr *gene expression increased, whereas *cra *gene expression decreased, and *icdA *and *mdh *gene expressions eventually decreased.

**Figure 4:**
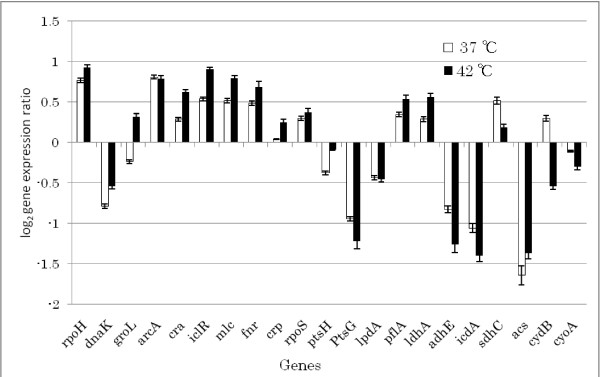
Effect of temperature on gene expressions of *E. coli *BW 25113 under microaerobic condition.

In order to see the effect of aeration, we have also conducted batch cultivations at microaerobic (or essentially anaerobic) condition. Table 3 shows the comparison of the yields at the time when glucose was depleted in the batch culture. Table 3 indicates that acetate, formate and lactate were more produced, while ethanol was less produced at 42°C as compared to the case at 37°C. Figure 4 indicates that *rpoH *gene expression increased at higher temperature, and the expressions of such genes as *dnaK *and *groL *followed the same pattern. The *arcA *gene expression changed little but *fnr *gene expression increased at higher temperature. The gene expressions of *cra, iclR *and *crp *increased at higher temperature. The expressions of *ptsG, icdA, sdhC, adhE *and *cyo *genes decreased, while *pflA *and *ldhA *expressions increased at higher temperature as compared to those at 37°C, which is consistent with the fermentation data of Table 3.

**Table 3:** Fermentation parameters for the microaerobic batch culture of wild type *Escherichia coli*.

Growth parameters	Culture temperature		% changes
		
	**37°C**	**42°C**	
Biomass yield (gDCW/g substrate)	0.12 ± 0.01	0.098 ± 0.02	- 18.3
Acetate yield (g/g substrate)	0.25 ± 0.03	0.32 ± 0.02	+ 28.0
Lactate yield (g/g substrate)	0.26 ± 0.02	0.31 ± 0.01	+ 19.2
Formate yield (g/g substrate)	0.25 ± 0.03	0.29 ± 0.02	+ 16.0
Succinate yield (g/g substrate)	0.02 ± 0.002	0.03 ± .003	+ 50.0
Ethanol yield (g/g substrate)	0.15 ± 0.01	0.13 ± 0.02	-1 3.3

± Standard deviation of three independent experiments.

## Discussion

To survive, cells have to control gene expressions precisely in response to the changes in the growth environment. The microorganism such as *E. coli *attains this primarily at the transcription level. To control the initiation of the specific transcription, the cell uses diverse mechanisms including various sigma factors. The classical heat shock regulon has been shown to be under the control of σ^32 ^transcription factor, the product of the *rpoH *gene [30]. The regulation of the sigma factor (σ^32^) is complex and depends on the feedback control loops involving the *dnaK *chaperone and temperature-induced changes in mRNA secondary structure [31]. The relative levels of the major heat shock genes such as *dnaK, groS, groL, ibpB, lonA *and *htpG *were found to be up-regulated after the temperature up-shift. The expressions of heat shock genes such as *dnaK, groL*, and *ibpB *increased in the early induction phase (first 10–20 minutes) and then declined (Fig. 2a). In *E. coli*, heat shock protein synthesis rates peak at about 5~10 min after the temperature up-shift and then declined to a new steady-state levels [32]. The heat shock response is made transcriptionally, where it has been known that the RNA polymerase core (E) binds to new initiation subunit σ^32 ^[33], and the resulting holoenzyme Eσ^32^, transcribes only heat shock genes [1], which have promoter sequences that differ from those transcribed by E plus σ^70^, the normal vegetative initiation factor [34]. The transcription factor σ^70 ^is itself a heat shock protein and the increase in its concentration after heat shock may contribute to its decline in heat shock protein synthesis. Moreover, other heat shock proteins, in particular the *dnaK *gene product contributes to the shutoff, since the mutations in their genes prolong the high level synthesis of heat shock proteins [35]. The heat shock response must be tightly regulated in order to allow rapid changes in heat shock protein synthesis rates. Although the level of mRNA transcribed from the *rpoH *gene increases after heat shock, their increases may be insufficient and too slow to be the sole explanation of the rapid effect of the heat shock. It has been shown that the concentration of active σ^32 ^limits the expression of heat shock genes, and that the stability of σ^32 ^is modulated [32].

Because of the rapid turn-over (half life of less than 1 min), the cellular concentration of σ^32 ^is very low at normal temperature and is limiting for the transcription of the heat shock gene. Upon temperature up-shift, σ^32 ^becomes transiently stabilized until the beginning of the shut-off phase of the heat shock response. The heat shock response is induced as a consequence of declining σ^32 ^levels and inhibition of σ^32 ^activity. Stress- dependent changes in heat shock gene are mediated by the antagonistic action of σ^32 ^and negative modulators which act upon σ^32^. These modulators are the DnaK chaperone system which inactivates σ^32 ^by direct association and mediates its degradation by proteases [36]. Degradation of σ^32 ^is mediated mainly by FtsH, and ATP dependent metallo- protease within the inner membrane. The heat shock proteins increased immediately after the temperature up-shift, reached a maximum 5–15 min later, and decreased to preshift values largely within 1 h, while the maximum induction of many heat-shock proteins including DnaK and HtpG reached at least 30 min later.

The *E. coli *cyclic AMP (cAMP) receptor protein Crp activates transcription for more than 100 promoters. When bound to its allosteric effector cAMP, the Crp homodimer binds to the specific DNA sites near target promoters, enhancing the binding of RNA polymerase holoenzyme (RNAP), and facilitating the initiation of the transcription. The present result indicates that *crp *gene expression increased and *lpdA *gene expression followed the similar pattern, but the effect of these gene expressions may not be significant for the change in the metabolism, or not clear at this time.

The present result shows that *mlc *gene expression followed the same pattern as that of *rpoH *upon heat shock, which confirms that Eσ^32 ^is involved in the expression of *mlc *gene. It has been shown that Eσ^32 ^plays an important role in balancing the relative concentration of Mlc and EIICB in response to the availability of glucose in order to maintain inducibility of Mlc regulon at higher temperature [28]. When Mlc was overproduced, it has been known to reduce acetate accumulation [37], and causes slow growth but gives better performance for recombinant protein production [38]. Mlc is a global regulator of carbohydrate metabolism, and regulates the expression of *pts *operon (Additional file 2). It has been known that Mlc represses *manXYZ *encoding enzyme II of the mannose PTS [39], *malT *encoding the activator of maltose operon, and *mlc *itself negatively [40]. Moreover, *ptsG *encoding enzyme IICB of the glucose PTS (EIICB^glu^) and the *pts *operon encoding general PTS proteins are also known to be repressed by Mlc [41,42]. The *mlc *promoter is very weak because nucleotide sequence of -10 region of the promoter differs from the consensus sequence of the strong promoter of *E. coli*. In addition, Mlc expression is autoregulated by Mlc itself. Therefore, the intracellular concentration of Mlc is limited in *E. coli *[43]. The *mlc *gene has been known to be transcribed by two promoters, P1 and P2, and have a binding site of its own gene product. It has been shown by in vitro transcription assays of *mlc *gene that P2 promoter could be recognized by RNA polymerase containing the heat shock sigma factor σ^32 ^(Eσ^32^) as well as Eσ^70^, while P1 promoter is only recognized by Eσ^70^. Figures 1 and 4 indicate that *ptsG *gene expression decreased (p < 0.05) in accordance with the up-regulation of *mlc *gene expression, while *ptsH *did not follow such pattern, where *ptsH *is not under control of Mlc and it was activated partly due to down-regulation of *cra *gene expression (Additional file 2).

Let us consider the production mechanism of acetate at higher temperature. In the typical batch cultivation, the cells must switch efficiently from the rapid growth on a favored carbon source such as glucose to a much slower growth on the excreted by-products such as acetate. Acetate excretion occurs through the phosphotransacetylase-acetate kinase (Pta-Ack) pathway, or may possibly by Pox (pyruvate oxidase) pathway. Acetate utilization occurs through AcCoA synthetase (Acs). This high- affinity acetate- scavenging enzyme converts acetate to AcCoA, where cells introduce it into the TCA cycle to generate energy and/or the glyoxylate pathway to build cell constituents. The higher expression of *acs *accelerates acetate assimilation in the presence of acetate [44,45], which leads to the activation of glyoxylate pathway. Transcription occurs from two σ^70^- dependent promoters such as the distal promoter *acs *P1 and proximal promoter *acs P2 *[44,46]. While multiple factors influence transcription, Crp appears to function directly as the critical transcription factor. Cells control this acetate switch primarily by controlling the initiation of *acs *transcription from the major promoter *acsP2 *[44,47]. Activation of *acs *transcription depends on the cAMP-Crp. The cAMP-Crp binds two sites within the *acs *regulatory region. However, it has been shown that Fis and Ihf independently modulate Crp- dependent activation of *acs*P2 transcription [48], and the mechanism is not so simple. As such, the activation of *crp *may cause *acs *to be up-regulated (Figs. 1 and 4). The *acs *gene is also under control of *rpoS*. In our previous investigation, it was shown that *acs *was expressed in an *rpoS*- dependent manner during different phases of the batch growth [49], but *rpoS *did not change much in the present case as shown in Figs. 1 and 4.

Although cellular ATP may increase for short period after the temperature up-shift in *E. coli *[50], it eventually decreases at higher temperature [6,50]. It has also been reported that the specific CO_2 _production rate as well as O_2 _consumption rate increased upon temperature up-shift [6,50,51]. As a result, the cell yield decreased and the cell maintenance increased [6,52]. Although it has been reported that the TCA cycle flux increased upon temperature up-shift at the specific growth rate of 0.08 h^-1^[50], recent investigation based on ^13^C- labeled experiment indicates that the TCA cycle flux became low at the dilution rates of 0.45 and 0.32 h^-1^[53]. Our result implies the repression of TCA cycle genes due to up-regulation of *arcA *gene, while the respiratory activity became higher due to up-regulation of *cyd *gene, which may be caused by the up-regulation of *arcA *gene. The *icdA *and *aceA *genes are known to be repressed by ArcA/B and activated by Cra (Additional file 2). The up-regulation of *arcA *gene expression and down regulation of *cra *gene expression both acted to repress *icdA *and *aceA *genes, and thus the TCA cycle as well as the glyoxylate pathway was repressed. In accordance with this, the *fadR *and *iclR *expressions were also up-regulated, which are known to repress *aceBAK *operon. This may have caused more acetate accumulation. Moreover, the down-regulation of *cyoA *may have limited the respiratory activity, while it may be counteracted by the activation of *cydB *gene. It has been reported that the respiration was activated during the temperature up-shift [6]. It may be due to the activation of *cydB *gene expression since K_m _value is lower or the affinity to oxygen is higher for Cyd as compared to Cyo. The global regulator, *arcA *showed increased expression after the temperature up-shift (especially first 30 mins) and modulated the expressions of such genes as *cydB, cyoA, icdA *etc. The up-regulation of *arcA *gene may not be the direct effect of heat shock but indirectly due to lower dissolved oxygen concentration caused by the lower solubility at higher temperature [50].

The down-regulation of *cra *gene expression may not be direct but may be due to the increase in the glucose concentrations in the fermentor, since the glucose uptake rate was reduced at higher temperature (Table 1). It has been reported that superoxide dismutase gene (*sod*) is induced in response to the oxidative stress imposed by dioxygen or by the redox active compounds such as viologens or quinones caused by the temperature up-shift [54]. It has also been reported that the exposure of a *sodA/B *null mutant *E. coli *to aerobic heat stress caused a profound loss of viability [55]. However, our experimental data of Fig. 1 indicate little change in *sod *expression. This may be due to the fact that the temperature was changed from 37°C to 42°C in the present experiment, while above experiment[52] was conducted at 45–48°C. Moreover, the *sod *gene is under control of SoxRS, where it becomes significant under dual osmotic and heat stresses [56].

**Figure 5:**
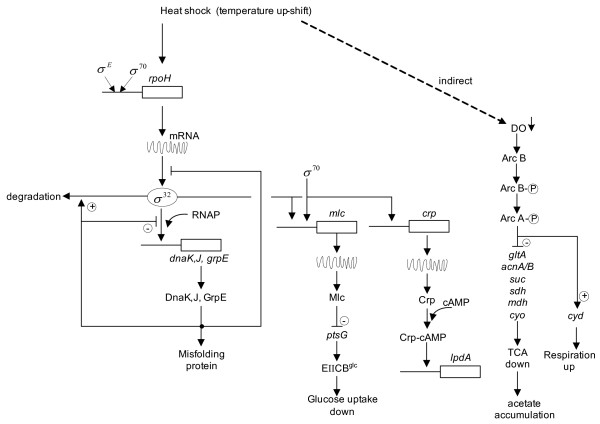
Schematic diagram of the effect of heat shock on gene expressions and metabolic regulation.

The common obstacle for the efficient metabolite production is the formation of by-products such as acetate. The present result suggests to use *arcA *gene knockout mutant. However, *arcA *gene knockout reduces the cell yield due to the activation of the TCA cycle. The cell yield can be increased by further knockout of such genes as *fadR *[57] or *iclR *[58]. In some cases, the dilution rate was lowered at the time of temperature up-shift in order to reduce the acetate formation, but the comparison must be careful in such a situation due to both effects of temperature up-shift and lower dilution rate. Moreover, in the case of induction of heterologous proteins, the result must be carefully interpreted, since both higher temperature and hetrologous protein production impose stress to the cell [59]. The present results do not contain such factors as different dilution rate or heterologous protein production, and it was clarified how the metabolism changed by the temperature up-shift in view of gene expressions. The overall mechanism may be illustrated as Fig. 5, where the left half part of heat shock regulation is already known [36], while the right half part was found in the present investigation.

## Conclusion

As shown in Fig. 5, the present research result clarified the mechanism of metabolic changes upon temperature up-shift from 37°C to 42°C based on gene expressions of heat shock genes, global regulators, and the metabolic pathway genes. In particular, the temperature up-shift caused the up-regulation of *mlc *gene, which in turn caused the down-regulation of *ptsG *gene expression, and finally caused the decreased in the specific glucose up-take rate. It was also found that the temperature up-shift also caused the up-regulation of *crp *gene expression, and activated *lpdA *gene expression. Moreover, it was shown that the temperature up-shift caused other TCA cycle genes to be repressed due to up-regulation of *arcA *gene indirectly caused by lower dissolved oxygen concentration and caused higher acetate production. This information is useful for the variety of applications such as temperature-induced heterologous protein productions, SSF etc. In particular, it is suggested to use *arcA *gene knockout mutant to reduce the acetate overproduction caused by the temperature up-shift with some careful consideration of its effect on the cell yield.

## Materials and methods

### Bacterial strains and culture conditions

The strains used were Escherichia coli BW25113 (*lacI*^q ^rrnB_T14 _Δ*lacZ*_WJ16 _*hsdR514 *Δ*araBAD*_AH33 _Δ*rhaBAD*_LD78_), and its arcA gene knockout mutant (JW4364). Batch and continuous cultivations were conducted using 2- l jar fermentor (M-100, Rikakikai Co. Tokyo, Japan), where the temperature was kept constant either at 37°C or 42°C, and the pH of the culture was maintained at 7.0 ± 0.1 by addition of 2.0 M HCl or 2.0 M NaOH with a pH controller. The air flow rate was maintained at 1 liter min-1 and the agitation speed was 350 rpm to ensure dissolved oxygen level to be about 40% of air saturation in the aerobic cultivation. The microaerobic cultivation was initiated by 2 h of aerobic cultivation followed by the microaerobic cultivation without supplying air and slowing down the agitation speed to around 100 rpm so that the cultivation was nearly anaerobic. Since the outlet of the fermentor was open to the atmosphere via the liquid bottle for mist trapping, the produced CO2 in the fermentor flowed out without increasing the overhead pressure. CO2 and O2 concentrations were measured by the off-gas analyzer (ABLE Co., Japan). The M9 synthetic medium was used, where it contained 10 g of glucose per liter, 48 mM Na_2_HPO_4_, 22 mM KH_2_PO_4_, 10 mM NaCl, and 30 mM (NH_4_)_2_SO_4_. The following components were filter sterilized and then added (per liter of final medium): 1 ml of 1 M MgSO_4_, 1 ml of 0.1 mM CaCl_2_, 1 ml of 1 mg of Vitamin B1 per liter, and 10 ml of trace element solution containing (per liter) 0.55 g of CaCl_2_, 1 g of FeCl_3_, 0.1 g of MnCl_2_.4H_2_O, 0.17 g of ZnCl_2_, 0.043 g of CuCl_2_.2H_2_O, 0.06 g of CoCl_2_.6H_2_O and 0.06 g Na_2_MoO_4_.2H_2_O. Continuous cultivations were performed at the dilution rate of 0.2 h^-1^, where the culture temperature was raised from 37°C to 42°C after the steady-state was ascertained.

### Measurements of biomass and extracellular metabolite concentrations

Cell concentration was measured by the optical density (OD) of the culture broth at 600 nm wave length with a spectrophotometer (Ubet-30, Jasco Co., Tokyo, Japan), and then converted to dry cell weight (DCW) per liter based on the relationship between OD and DCW previously obtained. Glucose concentration was measured using enzymatic kit (Wako Co., Osaka, Japan). Acetate, formate, lactate, succinate, and ethanol concentrations were also measured using enzymatic kits (Boehringer Co., Mannheim, Germany).

### RNA isolation, cDNA synthesis and PCR amplification

The 2.5 μl of culture broth was immediately suspended with 5 μl RNAprotect bacteria reagent. The samples were kept on ice for 5 minutes. After centrifugation at 10,000 rpm (4°C, 10 min), the supernatant was discarded and the pellet was stored at -80°C until RNA isolation. Total RNA was isolated from *E. coli *cells by Qiagen RNeasy Mini Kit (QIAGEN K.K., Japan) according to the manufacturer's recommendation. The quantity and purity of the RNA were determined by the optical density measurements at 260 and 280 nm and by 1% formaldehyde agarose gel electrophoresis. The sequences of the primers used in the present study are given elsewhere [60] except the followings:

*mlc *5' AGCAGACCAACGCGGGCGCG 3'

5' GACTATACGCAGGAAGGGCC 3'

Criteria for the design of the gene-specific primer pairs were followed according to Sambrook and Russel [61]. The primers used in this study were synthesized at Hokkaido System Science Co. (Sapporo, Hokkaido, Japan). In all cases, the primer-supplied company confirmed the absolute specificity of the primers.

RT-PCR reactions were carried out in a TaKaRa PCR Thermal Cycler (TaKaRa TP240, Japan) using Qiagen One Step RT-PCR Kit (QIAGEN K.K., Japan). The reaction mixture was incubated for 30 min at 50°C for reverse transcription (cDNA synthesis) followed by 15 min incubation at 95°C for initial PCR activation. Then the process was subjected to 30 cycles of amplification which consisted of a denaturing step (94°C for 1 min), an annealing step (approximately 5°C below melting temperature, of primers for 1 min) and an extension step (72°C for 1 min), and finally the reaction mixture of 25 μl was subjected for 10 min at 72°C for final extension. To check for nucleic acid contamination, one negative control was run in every round of RT-PCR. This control lacks the template RNA in order to detect possible contamination of the reaction components. 5 μl of amplified products were run on 1.8% agarose gel. Gels were stained with 1 mg ml^-1 ^of ethidium bromide, photographed using a Digital Image Stocker (DS-30, FAS III, Toyobo, Osaka, Japan) under UV light and analyzed using Gel-Pro Analyzer 3.1 (Toyobo, Osaka, Japan) software. In order to determine the optimal amount of input RNA, the two-fold diluted template RNA was amplified in RT-PCR assay under identical reaction condition to construct a standard curve for each gene product. When the optimal amount of input RNA was determined for each gene product, RT-PCR was carried out under identical reaction condition to detect differential transcript levels of genes. The gene *dnaA*, which encodes *E. coli *DNA polymerase and is not subjected to variable expression, i.e. abundant expression at relatively constant rate in most cells, was used as an internal control in the RT-PCR determinations. The gene expressions are given as relative values to that of *dnaA*. To calculate the standard deviation, RT-PCR was independently performed three times under identical reaction condition.

To ensure that the observed expression changes were statistically significant, the Student's t-test was applied.

## Competing interests

The authors declare that they have no competing interests.

## Authors' contributions

Monirul Hasan: All experiments. Kazuyuki Shimizu: Experimental design and manuscript preparation.

## Supplementary Material

Additional file 1 **Five tables.** Click here for file

Additional file 2 **Global regulators and its regulated genes.** Click here for file
