# Supplementary material for: Effect of temperature up-shift on fermentation and metabolic characteristics in view of gene expressions in Escherichia coli
Source: Microb Cell Fact. 2008 Dec 2;7:35. doi: 10.1186/1475-2859-7-35 (PMC2634768; doi:10.1186/1475-2859-7-35)
Supplement: Additional file 1 — Five tables. [file 1475-2859-7-35-S1.doc]

1. Glycolysis and Pentose phosphate pathway genes

| Gene name | Protein name | B number* | Description |
| --- | --- | --- | --- |
| *ptsG* | IICB ^glu^ | b1101 | Enzyme IIBC(Glc) |
| *ptsH* | PtsH | b2415 | Phosphohistidinoprotien-hexose phosphotransferase |
| *pykF* | Pyk | b1676 | Pyruvate kinase |
| *zwf* | Zwf | b1852 | Glucose6-phosphate dehydrogenase |

*Unique identifier for *E. coli* genes.

1. TCA cycle, glyoxylate pathway, and respiratory chain gene

| Gene name | Protein name | B number* | Description |
| --- | --- | --- | --- |
| *aceA* | AceA | b4015 | Isocitrate lyase |
| *ackA* | Ack | b2296 | Acetate kinase |
| *acs* | Acs | b4069 | AcCoA synthetase |
| *acnB* | Acn | b0118 | Aconitase B |
| *cyoA* | CyoA | b0432 | Cytochrome o oxidase |
| *cydB* | CydB | b0734 | Cytochrome d oxidase |
| *fumA* | Fum | b1612 | Fumerase |
| *gltA* | GltA | b0720 | Citrate synthase |
| *icdA* | IcdA | b1136 | Isocitrate dehydrogenase |
| *lpdA* | Lpd | b0116 | Lipoamide dehydrogenase |
| *mdh* | Mdh | b3236 | Malate dehydrogenase |
| *sucA* | SucA | b0726 | **α-**keto gluterate dehydrogenase |
| *sdhC* | Sdh | b0721 | Succinate dehydrogenase membrane anchor subunit |

*Unique identifier for *E. coli* genes.

1. Fermentative pathway genes

| Gene name | Protein name | B number* | Description |
| --- | --- | --- | --- |
| *adhE* | Adh | b1241 | Alchol dehydrogenase |
| *ldhA* | Ldh | b1380 | D-lactate dehydrogenase |
| *pflA* | Pfl | b0902 | Pyruvate formate lyase |
| *poxB* | Pox | b0871 | [Pyruvate oxidase](http://BioCyc.org/ECOO157/NEW-IMAGE?type=ENZYME&object=POXB-MONOMER) |

*Unique identifier for *E. coli* genes.

1. Heat shock and related genes

| Gene name | Protein name | B number* | Description |
| --- | --- | --- | --- |
| *dnaK* | DnaK | b0014 | Hsp70 molecular chaperone |
| *groL* | GroL | b4143 | Chaperonin Cpn60 |
| *groS* | GroS | b4142 | Chaperonin Cpn10 |
| *htpG* | HtpG | b0473 | Heat shock chaperone |

*Unique identifier for *E. coli* genes.

1. Global regulators

| Gene name | Protein name | B number* | Description |
| --- | --- | --- | --- |
| *arcA* | ArcA | b4401 | Anoxic redox control |
| *cra* | Cra | b0080 | Catabolite repressor/activator |
| *crp* | Crp | b3357 | [cyclic AMP receptor protein](javascript:if(window.name=='') { window.location.href='./nil'; } else { doaction(null, 517105, 126398); }) |
| *fnr* | Fnr | b1334 | Fumarate and nitrate reductase |
| *fadR* | FadR | b1187 | [Fatty acid degradation regulator](javascript:if(window.name=='') { window.location.href='./nil'; } else { doaction(null, 466146, 60957); }) |
| *iclR* | IclR | b4018 | Isocitrate lyase repressor |
| *mlc* | Mlc | b1594 | [Making large colonies](javascript:if(window.name=='') { window.location.href='./nil'; } else { doaction(null, 168624, 60956); }) |
| *rpoS* | RpoS | b2741 | [RNA polymerase sigma factor](javascript:if(window.name=='') { window.location.href='./nil'; } else { doaction(null, 170304, 60956); }) |
| *soxS* | SoxS | b4062 | Regulatory protein of soxRS regulon |

*Unique identifier for *E. coli* genes.
