# Supplementary material for: Effect of temperature up-shift on fermentation and metabolic characteristics in view of gene expressions in Escherichia coli
Source: Microb Cell Fact. 2008 Dec 2;7:35. doi: 10.1186/1475-2859-7-35 (PMC2634768; doi:10.1186/1475-2859-7-35)
Supplement: Additional file 2 — Global regulators and its regulated genes. [file 1475-2859-7-35-S2.doc]

Global regulators and its regulated genes

ArcA/B: *aceBAK-; aceEF-; acnA-; fumAC-; gltA-; icdA-; lpdA-; mdh-; ptsG-; sdhCDAB-;pfl+, cyd+, cyo-*

Cra: *aceA+; acnA+;acnB-; eda-;edd-; eno-; fbp+; gapA-, icdA+: pckA+; pfkA-, pps+; ptsHI-; pykF-*

Crp/Cya: *aceAB+; aceE+; acnAB+; crr+; fumA+; gltA+; lpdA-; mdh+; pckA+; ptsG+; ptsHI+; sdhABCD+; sucABCD+; tpiA+*

Fnr: *acnA-; fumAC-; icdA-; lpdA-; ptsG-; sdhCDAB-; talA-, frd+, pfl+*

Mlc: *crr-; ptsG-; ptsHI-;manXYZ-;malT-*
